# On the ‘border-lines’ of ADHD: Personality Traits, Diagnostic Overlaps and Clinical Implications

**DOI:** 10.1192/j.eurpsy.2025.754

**Published:** 2025-08-26

**Authors:** G. Soares, A. Lourenço, A. L. Falcão, I. Mateus, M. Nascimento, C. Oliveira

**Affiliations:** 1 Clínica 3, Hospital Júlio de Matos - ULS São José, Lisboa, Portugal

## Abstract

**Introduction:**

Attention Deficit and Hyperactivity Disorder (ADHD) is a neurodevelopmental condition characterized by persistent patterns of inattention, hyperactivity, and impulsivity. Beyond these core symptoms, ADHD seems to present complex associations with certain personality traits and to share several clinical features with personality disorders (PDs), particularly those within Cluster B. This overlap of symptomatology may lead to diagnostic challenges and potential misdiagnoses. This paper reviews the literature on the relationship between ADHD and personality traits, highlighting overlaps with personality disorders and exploring their clinical and diagnostic implications.

**Objectives:**

The primary objective of this review is to understand the potential associations between ADHD and specific personality traits, focusing on the extent to which these traits overlap with clinical features of personality disorders.

**Methods:**

A non-systematic literature review was conducted using major databases such as PubMed, Wiley and ScienceDirect targeting peer-reviewed studies published over the last two decades. The search terms included “ADHD,” “personality traits,” “personality disorders,” and “diagnostic overlap.” Relevant studies were selected based on their focus on adult ADHD and its association with personality traits and personality disorders. Review articles and cross-sectional studies were included.

**Results:**

The currently available literature reveals significant associations in the clinical presentation of ADHD and specific personality traits (changing accordingly to different models of personality assessment), as well as a relevant diagnostic overlap with Cluster B personality disorders, particularly Borderline Personality Disorder (BPD) and Antisocial Personality Disorder (ASPD). Shared features include impulsivity, emotional dysregulation, and difficulty in maintaining relationships. The presence of ADHD seems to increase the likelihood of personality pathology, with some studies suggesting a high co-occurrence of ADHD with traits of increased neuroticism and novelty-seeking, and decreased conscientious inhibition.

**Conclusions:**

ADHD and personality disorders share multiple overlapping clinical features, making accurate diagnosis challenging and potentially delaying adequate treatment. Thus, as suggested in some of the articles reviewed, an integrative and dimensional approach to such clinical pictures may be more adequate, so to ensure a profound understanding of the difficulties presented by patients, aiming at providing accurate and tailored treatment. Further research is needed to refine diagnostic criteria and strengthen a standardized dimensional thinking to address this diagnostic ambiguity.

**Disclosure of Interest:**

None Declared

